# Pasta Enrichment with an Amaranth Hydrolysate Affects the Overall Acceptability while Maintaining Antihypertensive Properties

**DOI:** 10.3390/foods8080282

**Published:** 2019-07-24

**Authors:** Eduardo Enrique Valdez-Meza, Anabela Raymundo, Oscar Gerardo Figueroa-Salcido, Giovanni Isaí Ramírez-Torres, Patrícia Fradinho, Sonia Oliveira, Isabel de Sousa, Miroslava Suárez-Jiménez, Feliznando Isidro Cárdenas-Torres, Alma Rosa Islas-Rubio, Guillermo Rodríguez-Olibarría, Noé Ontiveros, Francisco Cabrera-Chávez

**Affiliations:** 1 Department of Research and Food Science, University of Sonora, Hermosillo, Sonora 1658, Mexico; 2 LEAF-Linking Landscape, Environment, Agriculture and Food, Instituto Superior de Agronomia, Universidade de Lisboa, Tapada da Ajuda, 1349-017 Lisboa, Portugal; 3 Nutrition Sciences Academic Unit, University of Sinaloa, Culiacán, Sinaloa 80019, Mexico; 4 Department of Chemical and Biological Sciences, University of Sonora, Hermosillo, Sonora 83000, Mexico; 5 Research Center for Food and Development, Hermosillo, Sonora 83000, Mexico; 6 Division of Sciences and Engineering, Department of Chemical, Biological and Agricultural Sciences, University of Sonora, Navojoa, Sonora 85880, Mexico

**Keywords:** amaranth protein, pasta, sensory evaluation, hypertension, functional food

## Abstract

Background: Alcalase-treated amaranth proteins generate angiotensin-1-converting enzyme (ACE-1) inhibitory peptides, which could be useful for functional foods development. Our aim was to evaluate the technological, sensory, and antihypertensive properties of pasta enriched with an amaranth hydrolysate. Methods: Pasta with 11% (A; control), 15% (B), and 20% (C) of protein content were formulated. Pastas B and C were supplemented with an alcalase-treated amaranth protein concentrate. Cooking time, cooking lost, color, and texture were assessed. An untrained panel (*n* = 30) evaluated sensory attributes. The antihypertensive effect was evaluated in hypertensive rats. Results: The hydrolysate IC50 was 0.014 mg/mL. Optimum cooking time and cooking loss decreased in products B and C vs. A (*p* < 0.05). The L* values decreased in pasta C. Firmness increased in pasta C vs. A (*p* < 0.05). Adhesiveness was different among groups (*p* < 0.05). Pasta A had the highest acceptability (*p* < 0.05). The products B and C, and captopril (positive control) showed antihypertensive properties after 3 h of supplementation (*p* < 0.05). This effect remained after 7 h, 8 h, or 9 h. Conclusions: The addition of amaranth hydrolysates to pasta negatively impacts on the overall acceptability and, to a lesser extent, on pasta taste. However, it is possible to maintain the antihypertensive properties of the supplemented pasta under physiological conditions.

## 1. Introduction

Functional foods provide health-related benefits beyond basic nutrition by virtue of the presence of physiologically active components [1,2]. These foods could contribute as an adjunct for the control of several diseases, such as high blood pressure. Potential ingredients for this purpose include anthocyanins [3], isoflavones [4], and peptides [5]. The mechanism for reducing blood pressure involves several pathways. Pharmaceutical drugs such as enalapril and captopril are inhibitors of angiotensin-1-converting enzyme (ACE-1), which is involved in the pathogenesis of hypertension. This ACE-1 inhibitory activity is also exhibited by peptides from several dietary sources [6,7,8]. Particularly, di- and tri-peptides are absorbed more rapidly than free amino acids, mainly through the paracellular and transcellular routes or by specific transporter systems, such as the peptide transporter PepT1 [9,10]. Recent works have proven that the hydrolysis of amaranth proteins with microbial alcalase can release ACE-1 inhibitory peptides [11], and it has been proposed that both the bioavailability and the antihypertensive properties of the bioactive peptides are influenced by the food matrix characteristics [9]. For instance, the bioavailability of antihypertensive peptides can be improved by increasing the protein content of the food matrix, mainly due to delay on the gastric emptying [12].

The technological properties of food matrices, as well as their sensory characteristics, could be modified after the incorporation of new ingredients into standardized formulations. In fact, the incorporation of microalgae biomass [13] or amaranth proteins [14] in food pasta formulations modifies the technological and sensory profile of cooked pasta. This highlights that the addition of bioactive compounds to standardized pasta formulations deserves the evaluation of the technological and sensory properties of the new supplemented food product. Furthermore, the health benefits claimed should be evaluated in vivo to support, at least in part, the functionality of the functional foods proposed. Thus, the aim of the present study was to assess the technological and sensory characteristics of pasta, formulated with different protein content and supplemented with an amaranth hydrolysate that has shown antihypertensive properties in vivo [11], as well as the suitability of the developed pasta for lowering systolic blood pressure in spontaneously hypertensive rats.

## 2. Materials and Methods

### 2.1. Amaranth Protein Hydrolysis

The amaranth protein hydrolysate was obtained as previously described with minor modifications [11]. Amaranth protein concentrate (COPRAM^TM^) was suspended in purified water (1:5 *w*/*v*). The hydrolysis was carried out with food-grade alcalase (0.04 mU/mg of protein content) at 52 °C with continuous stirring for 6.16 h. The enzymatic hydrolysis was stopped by heating the reaction solution (85 °C, 15 min).

### 2.2. Half-Inhibitory Concentration

Half-inhibitory concentration (IC50) of the hydrolysate was assessed using an ACE-1 Inhibition kit (ACE kit-WST-Dojindo Molecular Technologies, Inc., Kumamoto, Japan) following the manufacturer’s instructions, where the substrate reaction was 3-Hydroxybutylyl-Gly-Gly-Gly. Serial dilutions of the amaranth hydrolysate were prepared and utilized to construct an ACE-1 inhibition curve. The protein concentration of the amaranth hydrolysate was determined using the BCA assay (BCA assay, Pierce^TM^ Thermo Scientific, Rockford, IL, USA). The concentration of amaranth hydrolysate required to produce 50% of ACE-1 inhibition was considered as the IC50 and was expressed as mg/mL.

### 2.3. Pasta Making

Ingredients for pasta making are listed in Table 1. Pasta with three different protein contents were prepared following the official American Association of Cereal Chemists (AACC) method 66-10.01 [15], with modifications in drying steps [16]. Protein concentrations were adjusted, adding amaranth protein concentrate (COPRAM™) to the standard pasta formulation (pasta A). The ingredients were mixed in a Kitchen Aid mixer (St Joseph, USA) according to the mixing time established in previous mixograms (10 min). The amaranth hydrolysate was gradually added during the mixing time until the final concentration was 4% (*w*/*w*). The mixture was extruded at room temperature using a pasta maker (Columbian Home Products #330-54, Terre Haute, IN, USA). The pasta products were dried in three steps: (1) 60 °C and 65% relative humidity for 3 h; (2) 40 °C and 30% relative humidity for 5 h (Micropak oven series MP500, Enviro-Pak, Clackamas, OR, USA); and (3) 50 °C for 3 h in an oven (RedLine RF 115 UL, Binder, Tuttlingen, Germany).

### 2.4. Proximate Composition

The proximate composition was evaluated according to Association of Official Analytical Chemists (AOAC) methods [17]. Moisture was assessed, drying the products at 105 °C for 24 h (method 925.09B); crude protein was measured using the micro-Kjeldahl assay (method 960.52); lipids were determined by defatting the products in a Soxhlet apparatus using petroleum ether as solvent (method 920.39C); ashes were obtained by incineration at 550 °C (method 923.03); and carbohydrates were calculated by difference of the other macronutrients. All evaluations were carried out in triplicate, and the results expressed in g/100 g dry basis.

### 2.5. Pasta Cooking Quality, Color, and Texture Evaluations

The technological characteristics of the pasta were evaluated according to the AACC method 66-10.01 [15]. Optimum cooking time was expressed in minutes; cooking loss and weight gain were expressed as g/100 g of pasta. The color parameters of both dried and cooked pasta were measured using a Minolta (Japan) digital colorimeter (CR-400). The results were expressed in the International Commission on Illumination (CIE) LAB space as L* (luminosity, 0 = black, 100 = white), a* (+a = red, −a = green) and b* (+b = yellow, −b = blue). The texture evaluations of cooked pasta were carried out according to the AACC method 74-09.01. Firmness (cutting strength) and adhesiveness were evaluated in cooked pasta using a TA-XTPlus texturometer (Stable Microsystems, Godalming, UK) under the following settings: load cell of 5 kg at test speed of 0.1 mm/s, distance of 1.6 mm, force threshold of 0.01 N, 3 cm strips of pasta. Seven evaluations per product were carried out. A force–distance curve was recorded in the texturometer, and maximum strength was considered as firmness (N). The negative area was recorded as adhesiveness (−N·s).

### 2.6. Sensory Evaluation

Thirty untrained panelists participated in the sensory analysis. Sensory attributes (color, odor, taste, and texture) and the global appreciation of the products were evaluated in a hedonic scale (0 = totally dislike, 10 = like very much) according to International Standard Organization (ISO)-11136:2014 [18]. A triangle test based on ISO-4120:2004 method [19] was carried out in order to verify the capability of the panelists to discriminate among the three types of pasta (A, B, and C). Each panelist was provided with six sequences of samples (1: A, A, B; 2: A, B, B; 3: A, A, C; 4: A, C, C; 5: B, B, C; and 6: B, C, C). The perceptible difference among the products was evaluated utilizing standardized tables, according to ISO-4120:2004.

### 2.7. In Vivo Evaluation of the Antihypertensive Properties of the Amaranth Hydrolysate

Groups of six spontaneously hypertensive male rats (12 weeks old, 300–350 g body weight) ate freely 8 g of cooked pasta A (control), B, or C. As a positive control, a fourth group of rats received captopril (25 mg/kg of rat body weight) intragastrically. Blood pressure was monitored before (time zero) and after treatments (at 1 h intervals for 9 h). The CODA™ tail cuff blood pressure monitor (Kent Scientific, Torrington, CT, USA) was utilized. An Ethics Review Board of the University of Sinaloa approved the study protocol (CE-UACNYG-2015-SEP-001).

### 2.8. Statistical Analysis

All data were analyzed using GraphPad Prism 6.0 (GraphPad Software, San Diego, CA, USA). Normality tests were carried out using Shapiro–Wilk test. Normally distributed data were expressed as mean and standard deviation. Differences among groups were analyzed using ANOVA and Tukey tests. Non-normally distributed data were expressed as median and interquartile range. Differences among groups were analyzed using Kruskal–Wallis and Dunn’s tests. A *p* value ≤ 0.05 was considered statistically significant.

## 3. Results

### 3.1. Amaranth Protein Hydrolysis and IC50 Estimation

**Figure 1:**
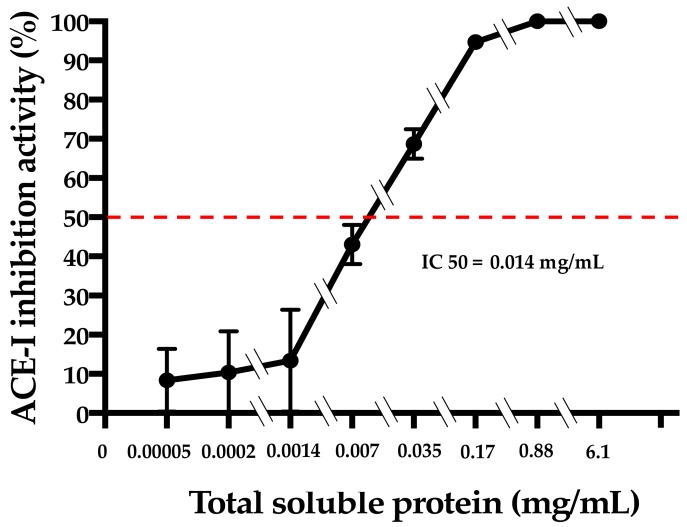
IC50 (half-inhibitory concentration) estimation of the amaranth hydrolysate (optimized hydrolysate parameters: pH 7.01; temperature 52 °C; enzyme concentration 0.04 mU/mg of protein; reaction time 6.16 h). ACE-1: angiotensin-1-converting enzyme.

The hydrolysis of amaranth protein with food-grade alcalase generates a hydrolysate with ACE-1 inhibitory activity. The IC50 value of the hydrolysate was 0.014 mg/mL, and 100% of ACE-1 inhibition was seen at 0.88 mg/mL (Figure 1).

### 3.2. Technological Evaluation of Pasta

The results of the proximate analysis are shown in Table 2. As expected, pasta C had the highest protein content (20% dry basis), followed by pastas B (15.0%) and A (11.1%). The optimum cooking time and cooking loss parameters, as well as the weight gain after cooking are shown in Table 3. Optimum cooking time and cooking loss decreased in the pasta with the highest protein content (C) (*p* ≤ 0.05). Weight-gain parameters were no different among the products (*p* > 0.05). Color variations in both dry and cooked pasta are shown in Table 4. After cooking, L* values decreased in pastas B and C (*p* ≤ 0.05), but not in the control pasta (A). Both a* and b* color parameters varied among the different types of pasta (*p* < 0.05). The development of a brown color was more evident in pastas B and C than in pasta A, either before or after cooking (*p* ≤ 0.05).

Texture was evaluated after cooking of pasta at optimum time. Adhesiveness was inversely correlated to protein content. On the contrary, the firmness of pasta increased as the protein content was augmented (*p* < 0.05) (Table 5).

### 3.3. Sensory Evaluation

**Figure 2:**
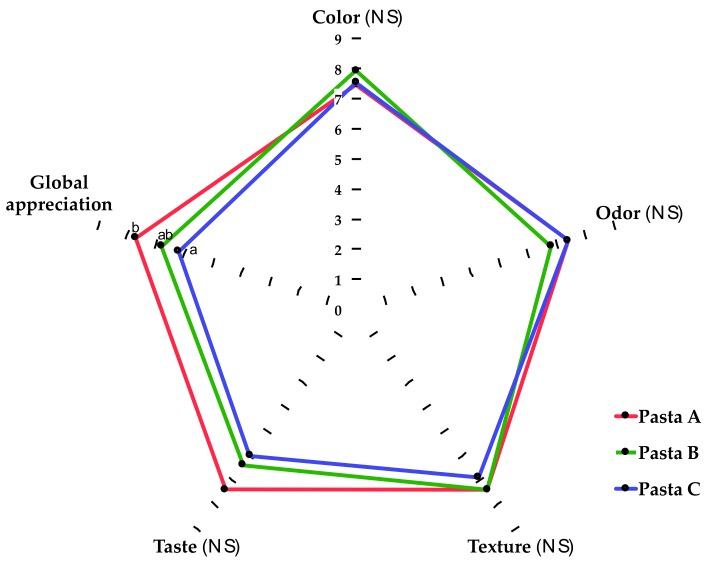
Sensory evaluation of cooked pasta. A hedonic scale was used, where 0 = totally dislike and 10 = like very much. Comparisons in each sensory attribute were carried out using ANOVA and Tukey tests. Different letters indicate statistical difference (*p* < 0.05). NS: nonsignificant difference.

**Figure 3:**
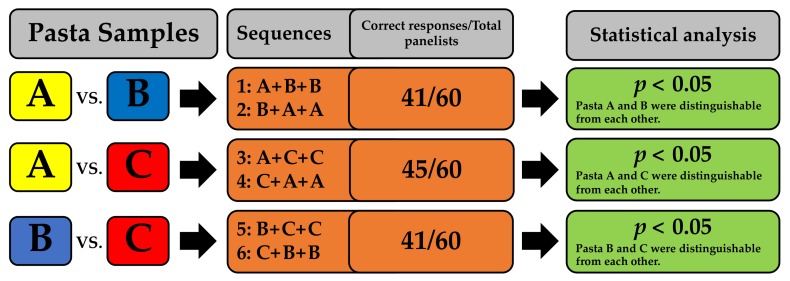
Triangle test to evaluate the capability of the panelists to discriminate among pasta samples. Sixty untrained panelists were recruited. Pastas (A, B, C) were assigned a code and presented simultaneously to all panelists. In each sample sequence, two equal pastas and one different pasta were given to panelists. Statistical differences were estimated according to ISO-4120:2004.

Pasta A (control pasta) scored better global appreciation and *taste* than pastas B and C, however, significant differences (*p* < 0.05) were observed for global appreciation only (Figure 2). Regarding color, odor, and texture, there were no significant differences (*p* > 0.05) among the three types of pasta. Triangle test was performed to evaluate if the panelists were able to distinguish one type of pasta from another. Overall, all the sequences of products evaluated were distinguishable from each other (*p* < 0.05) (Figure 3).

### 3.4. Supplemented Pasta Efficiently Reduces Systolic Blood Pressure in Spontaneously Hypertensive Rats

**Figure 4:**
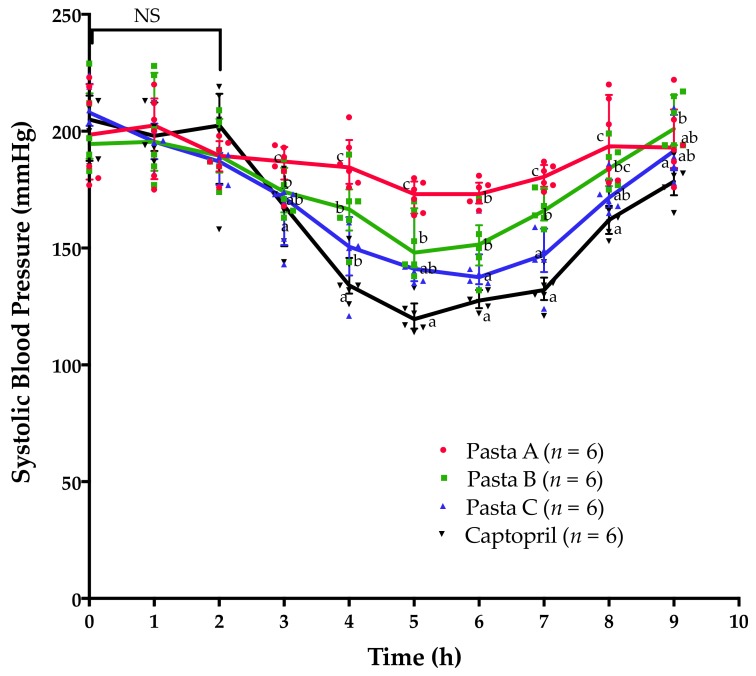
Systolic blood pressure in spontaneously hypertensive rats after supplementation with three types of pasta and captopril. Comparisons at each time point were carried out using nonparametric ANOVA and Kruskal–Wallis test. Different letters at specific time points mean significant difference (*p* < 0.05). NS: nonsignificant difference.

The baseline of blood pressure in spontaneously hypertensive rats was 199.33 ± 20.90 mmHg. Compared with control pasta, blood pressure was significantly reduced (*p* < 0.05) after 3 h of the ingestion of pasta B or C (both containing the amaranth hydrolysate) (Figure 4). The drug captopril showed more potent antihypertensive properties than the three types of pasta tested (*p* < 0.05) (Figure 4). Compared with control pasta, the blood pressure reduction was maintained for 7 h or 8 h in the groups supplemented with pasta B or C, respectively (*p* < 0.05). Captopril maintained its antihypertensive effect until the last evaluation of blood pressure, 9 h after captopril administration (Figure 4).

## 4. Discussion

In this study, the antihypertensive properties of an amaranth hydrolysate incorporated into a food matrix with different protein content were evaluated. The optimized hydrolysis conditions of amaranth protein with alcalase to generate an antihypertensive hydrolysate were previously established by our research group [11]. However, in this case, only food-grade reagents were utilized to hydrolyze amaranth protein. Although the IC50 value of the amaranth hydrolysate generated in this study (0.014 mg/mL) was higher than the IC50 value of captopril (0.0435E4 mg/mL; calculated by the manufacturer of the ACE-1 inhibition kit utilized), it was almost tenfold lower than the IC50 values reported by others who utilized the same enzyme and source of proteins [20]. This can be attributed to differences in the hydrolysis conditions. In fact, the hydrolysis degree of amaranth protein under the conditions used in the present study is 75% [9], while others reported hydrolysis degrees between 45% and 65% [20]. This is of relevance as di-, tri-, and tetra-peptides are the best ACE-1 inhibitory peptides, and these are preferentially generated after exhaustive proteolysis [9].

The antihypertensive peptides were added into pasta formulations with different protein content. The addition of protein proportionally reduces the starch content of the food matrix (Figure 2), and this reduction could impact on cooking time. As starch gelatinization during cooking is related to the optimum cooking time, this parameter decreased as the protein content of the pastas increased (Table 3). This is in line with previous studies [21]. On the other hand, cooking loss is related to the structural characteristics of the pasta matrix. Compared with conventional pasta formulation, cooking loss can either increase or decrease after the incorporation of additional ingredients [13,16]. This could be related to molecular rearrangements, which organize the microstructure of the matrix, making it more prone to tolerate cooking stresses [22]. A similar effect was previously reported in pastas enriched with microalgae biomass [13].

Regarding color analyses (Table 4), luminosity (L*) decreased in pastas as the protein content increased. The brown color developed in the pastas enriched with amaranth protein can be attributed to Maillard reactions, which occurred during pasta processing [23,24,25]. Maillard reactions require reducing sugars and free amino groups to occur. These amino groups could be more accessible in pastas supplemented with protein hydrolysates. The hydrolysate-containing pastas were also supplemented with nongluten proteins. Reducing the proportion of gluten proteins in the formulation commonly decreases the pasta firmness since the network can lose strength and the overall microstructure of the pasta is disrupted [26]. However, in the present study, the firmness of cooked pasta increased as the protein content increased as well. This improvement in firmness could be caused by the nature of the added protein and the heat treatment during drying steps, as previously reported [16].

Sensory assessment (Figure 2) indicated that color, odor, and texture values of the supplemented pastas were very close to the control pasta, but significant differences (*p* < 0.05) in the global appreciation were observed. The consumers did not find significant differences (*p* > 0.05) between the tastes of the pastas. Changes in sensory attributes were expected as the percentage of the ingredient (100 g of semolina) of the control pasta changed by 12.0% in pasta B (104.4 g total; 91.8 g of semolina) and by 45.2% in pasta C (104.0 g total; 57.0 g of semolina). This observation is based on a meta-analysis study, which indicates that enrichment levels above 10% significantly decrease the acceptance of supplemented pastas [27].

Systolic blood pressure of spontaneously hypertensive rats significantly decreased (*p* < 0.05) after 3 h of the ingestion of supplemented pastas compared with control pasta (Figure 4). Previous studies stated that orally administered antihypertensive peptides and captopril can reduce the blood pressure in spontaneously hypertensive rats, but the antihypertensive effect can be observed only after 3 h postsupplementation [28]. Our results support these findings and also show that similar to the oral administration of the amaranth hydolyzate per se [11], the most pronounced antihypertensive effects after the ingestion of amaranth-hydrolysate-supplemented pasta are observed between 5 h and 6 h. Furthermore, the results suggest that the protein content could play an important role for improving the antihypertensive performance of pasta enriched with amaranth hydrolysates. The antihypertensive effect was more evident in the group of animals fed with the supplemented pasta containing the highest proportion of protein (pasta C). Others have stated that food matrices with increased protein content can delay the release and absorption of functional compounds [12]. The data presented here and obtained in spontaneously hypertensive rats hardly support this notion, as the antihypertensive effect was lost after 9 h of the ingestion of any of the supplemented pastas. Furthermore, 8 h after the administration of the supplemented pastas, the antihypertensive effects were not statistically significant (*p* > 0.05). It can be hypothesized that the addition of protein induces molecular rearrangements of the food matrix microstructure [9], entrapping more efficiently the antihypertensive molecules generated during the digestion of amaranth proteins with alcalase. Supporting this, our results show that the addition of protein increases firmness, which can contribute to entrap the antihypertensive molecules in the food matrix during cooking.

In addition to the hydrolysate, the pastas evaluated were enriched with amaranth protein concentrate to increase their protein content. The simulated gastrointestinal digestion of amaranth protein concentrates generates ACE-1 inhibitory compounds [29], but the IC50 values reported for these hydrolysates are 31.1 fold higher than the IC50 value of the amaranth hydrolysate utilized in the present study. Similarly, the daily consumption of 0.38 g of amaranth protein concentrate incorporated into a food matrix can help to reduce blood pressure in rats after 4 weeks [30]. In our study and considering the weight gain of the pasta after cooking, the rats were fed only once with 8 g of cooked pasta, which is equivalent to 0.22 g or 1.14 g of amaranth protein concentrate, and the blood pressure measurements were carried out within the following 9 h. Under these experimental conditions, readily detected antihypertensive effects are not expected due to the intake of amaranth protein concentrate, which suggests that a food matrix enriched with the optimized amaranth hydrolysate obtained with alcalase significantly reduces the blood pressure in hypertensive rats after the first hours of its intake.

## 5. Conclusions

Amaranth hydrolysates obtained with alcalase have antihypertensive properties, which can be maintained after the hydrolysates incorporation into pasta. Moreover, the antihypertensive amaranth molecules incorporated maintain their bioavailability after pasta ingestion and efficiently reduce blood pressure in spontaneously hypertensive rats. Although some sensory attributes of the amaranth-hydrolysate-supplemented pasta need to be addressed in order to improve the consumer acceptance, the effectiveness of amaranth hydrolysates for lowering blood pressure justifies further studies to evaluate their safety and use as an ingredient for functional foods development.

## Figures and Tables

**Table 1:** Formulations for pasta making with changes in the content of protein *.

Ingredients (g)	Pasta A (11% Protein)	Pasta B (15% Protein)	Pasta C (20% Protein)
Semolina	100.00	91.87	57.00
Amaranth protein concentrate	-	8.53	43.00
Amaranth protein hydrolysate	-	4.00	4.00

* Based on the method 66-10.01 of the American Association of Cereal Chemists (AACC), (2001) [15].

**Table 2:** Proximate analysis of the three types of pasta (dry basis).

Sample	Moisture (g/100 g)	Protein (g/100 g)	Fat (g/100 g)	Ash (g/100 g)	Carbohydrates (g/100 g)
Pasta A	7.64 ± 0.01 ^a^	11.10 ± 0.01 ^a^	0.92 ± 0.61 ^a^	0.63 ± 0.01 ^a^	87.33 ± 2.04 ^c^
Pasta B	7.84 ± 0.01 ^a^	15.06 ± 0.29 ^b^	1.44 ± 0.34 ^b^	0.90 ± 0.01 ^a^	82.59 ± 0.34 ^b^
Pasta C	8.12 ± 0.01 ^a^	20.00 ± 0.06 ^c^	2.27 ± 0.40 ^b^	0.92 ± 0.01 ^a^	76.89 ± 0.21 ^a^

Comparisons in each column were carried out using ANOVA and Tukey tests. Different superscripts letters in the same column mean significant difference (*p* < 0.05). Mean values ± standard deviations are shown. Carbohydrates were calculated by difference.

**Table 3:** Cooking quality of the three types of pasta.

Sample	Optimum Cooking Time (MIN)	Cooking Loss (G/100 G)	Weight Gain (G/100 G)
PASTA A	10.5 ± 0.5 ^b^	11.5 ± 0.5 ^b^	207 ± 4.9 ^a^
PASTA B	7.5 ± 0.5 ^a^	8.9 ± 0.1 ^b^	205 ± 6.0 ^a^
PASTA C	5.5 ± 0.0 ^a^	7.3 ± 1.1 ^a^	193 ± 1.0 ^a^

Comparisons in each column were carried out using ANOVA and Tukey tests. Different superscript letters in the same column mean significant difference (*p* < 0.05). Mean values ± standard deviations are shown.

**Table 4:** Color parameters in dry and cooked pasta.

Sample	Presentation	L*	a*	b*
Pasta A	Dry	78.5 ± 0.7 ^d^	2.02 ± 0.1 ^a^	17.4 ± 0.8 ^b^
	Cooked	78.5 ± 1.6 ^d^	1.01 ± 0.6 ^a^	16.7 ± 0.9 ^a^
Pasta B	Dry	72.1 ± 0.5 ^c^	4.3 ± 0.2 ^b^	21.6 ± 0.5 ^c^
	Cooked	56.2 ± 1.2 ^a^	7.8 ± 0.2 ^c^	24.5 ± 0.5 ^de^
Pasta C	Dry	60.0 ± 0.3 ^b^	7.6 ± 0.2 ^c^	24.1 ± 0.3 ^d^
	Cooked	55.7 ± 0.6 ^a^	8.2 ± 0.2 ^d^	25.1 ± 0.4 ^e^

Comparisons in each column were carried out using ANOVA and Tukey tests. Different superscript letters in the same column mean significant difference (*p* < 0.05).

**Table 5:** Texture characteristics of the three types of pasta after cooking.

Sample	Firmness (N)	Adhesivness (−N·S)
Pasta A	3.31 ± 0.26 ^a^	0.053 ± 0.011 ^a^
Pasta B	3.64 ± 0.77 ^b^	0.043 ± 0.010 ^b^
Pasta C	5.73 ± 0.75 ^c^	0.041 ± 0.007 ^c^

Comparisons in each column were carried out using ANOVA and Tukey tests. Different superscript letters in the same column mean significant difference (*p* < 0.05). Mean values ± standard deviations are shown.
